# Suppression of APC/C^Cdh1^ has subtype specific biological effects in acute myeloid leukemia

**DOI:** 10.18632/oncotarget.10196

**Published:** 2016-06-21

**Authors:** Daniel Ewerth, Andrea Schmidts, Manuel Hein, Dominik Schnerch, Arunas Kvainickas, Christine Greil, Justus Duyster, Monika Engelhardt, Ralph Wäsch

**Affiliations:** ^1^ Department of Hematology, Oncology and Stem Cell Transplantation, University Medical Center, Faculty of Medicine, University of Freiburg, Freiburg, Germany; ^2^ Faculty of Biology, University of Freiburg, Freiburg, Germany; ^3^ Present address: Department of Cardiology and Angiology II, University Heart Center Freiburg - Bad Krozingen, Faculty of Medicine, University of Freiburg, Bad Krozingen, Germany; ^4^ Present address: Center for Biological Systems Analysis, University of Freiburg, Freiburg, Germany

**Keywords:** anaphase-promoting complex, Cdh1, ubiquitin-ligase, acute myeloid leukemia, differentiation

## Abstract

The E3 ubiquitin ligase and tumor suppressor APC/C^Cdh1^ is crucial for cell cycle progression, development and differentiation in many cell types. However, little is known about the role of Cdh1 in hematopoiesis. Here we analyzed Cdh1 expression and function in malignant hematopoiesis. We found a significant decrease of Cdh1 in primary acute myeloid leukemia (AML) blasts compared to normal CD34+ cells. Thus, according to its important role in connecting cell cycle exit and differentiation, decreased expression of Cdh1 may be a mechanism contributing to the differentiation block in leukemogenesis. Indeed, knockdown (kd) of Cdh1 in HL-60 cell line (AML with maturation, FAB M2) led to less differentiated cells and a delay in PMA-induced differentiation. Acute promyelocytic leukemia (APL, FAB M3) is an AML subtype which is highly vulnerable to differentiation therapy with all-trans retinoic acid (ATRA). Accordingly, we found that APL is resistant to a Cdh1-kd mediated differentiation block. However, further depletion of Cdh1 in APL significantly reduced viability of leukemia cells upon ATRA-induced differentiation. Thus, low Cdh1 expression may be important in AML biology by contributing to the differentiation block and response to therapy depending on differences in the microenvironment and the additional genetic background.

## INTRODUCTION

In the hematopoietic system balance between cell cycle progression on the one hand, and cell differentiation preceded by cell cycle exit on the other hand, is vital. Moreover, cell cycle control may be a reasonable target in acute myeloid leukemia (AML) [1, 2]. The anaphase-promoting complex/cyclosome (APC/C) is an E3 ubiquitin ligase that governs the cell cycle by targeting numerous cell cycle regulators for proteasomal destruction. Its coactivator Cdh1 is needed to establish a stable G0/G1 phase, which is an important precondition for precise cell cycle progression or differentiation and maintenance of genomic stability [3–8]. Thus, loss of Cdh1 may contribute to tumorigenesis by enhanced proliferation of undifferentiated and genetically unstable cells [9].

It has been shown in various models that APC/C^Cdh1^ establishes a stable G1/G0 phase by maintaining a low mitotic cyclin state [10–13] and degrading the F box protein Skp2, which leads to the stabilization of the SCF^Skp2^ targets and Cdk inhibitors p21 and p27 [14, 15]. In contrast, conditional inactivation of APC/C function causes quiescent G1/G0 mouse hepatocytes to re-enter the cell cycle [16]. APC/C^Cdh1^ also modulates TGFβ signaling by degrading the transcriptional regulators Klf4 and SnoN to induce target gene expression, which regulates growth inhibition and cell differentiation [17–19]. Other important APC/C^Cdh1^ targets to control the differentiation process are Id (inhibitor of differentiation) proteins [8].

A role of APC/C^Cdh1^ in the differentiation process has already been described in several cell types, such as neurons, myocytes, lens epithelial cells, hepatocytes and embryonic stem cells [16, 20–24]. However, little is known about the role of Cdh1 in the hematopoietic system. In order to study the role of APC/C^Cdh1^ in AML, we analyzed the protein expression patterns of Cdh1 in primary human AML blasts and the role of Cdh1 knockdown (kd) on induced differentiation in two cell lines derived from different AML subtypes using our previously validated highly efficient short hairpin (sh)RNA against Cdh1 [4, 25]. Cdh1 expression was decreased in the vast majority of primary AML samples. Further Cdh1 depletion contributed to a differentiation block in AML with maturation (FAB M2). On the contrary, acute promyelocytic leukemia (APL, FAB M3) with the unique t(15;17) translocation, where ATRA-induced differentiation is a highly efficient targeted treatment approach, was resistant to the Cdh1-kd effect on differentiation. However, viability of APL cells upon ATRA treatment was significantly reduced.

## RESULTS

### Cdh1 expression in primary AML samples

**Figure 1:**
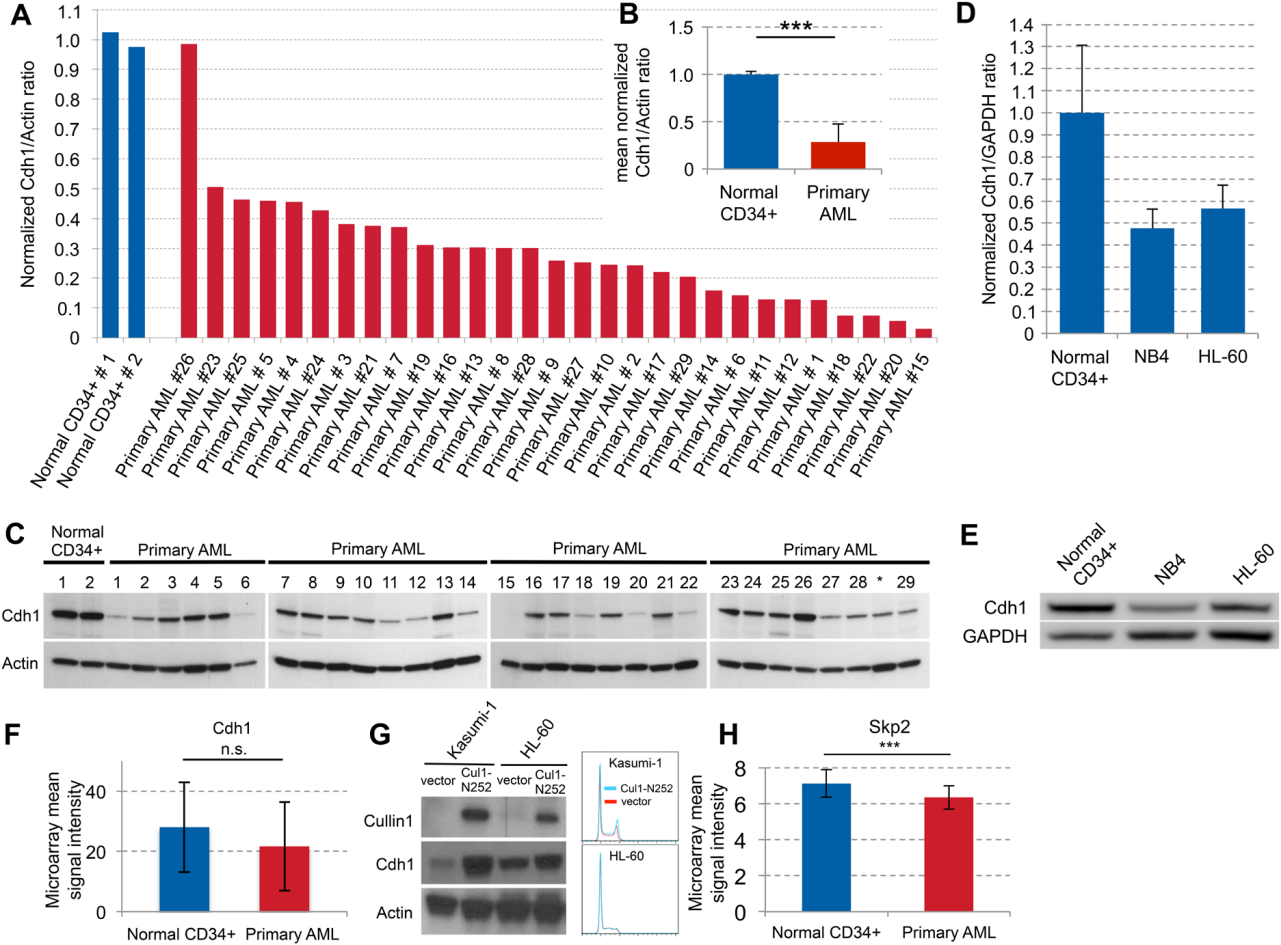
Cdh1 expression in primary AML samples and regulation in cell lines **A.** Normal CD34+ cells and samples from 29 AML patients were analyzed by western blot. Quantification of protein expression was used to determine Cdh1/Actin ratio and results were normalized to the mean of the 2 normal CD34+ samples. **B.** Normalized Cdh1/Actin ratio of primary AML samples presented as mean + s.d. p<0.001. **C.** Immunoblots for the indicated proteins as quantitated in (A and B). * Sample was excluded due to low blast count. **D.** Normal CD34+ cells and the AML cell lines NB4 and HL-60 were analyzed by western blot. Quantification of protein expression was used to determine Cdh1/GAPDH ratio and results were normalized to the mean of 3 different normal CD34+ samples. **E.** Immunoblots for the indicated proteins as quantitated in (D). **F.** Transcriptional analysis of Cdh1 expression in normal CD34+ and primary AML cells based on publicly available microarray dataset presented as mean +/− s.d. (GSE30029) [26]. n.s. - not significant. **G.** AML cell lines Kasumi-1 and HL-60 were lentivirally transduced with a construct to overexpress truncated Cullin 1 (Cul1-N252) or with empty vector, respectively and immunoblotted for the indicated proteins. Cell cycle histogramms are shown on the right. **H.** Transcriptional analysis of Skp2 expression in normal CD34+ and primary AML cells based on publicly available microarray dataset presented as mean +/− s.d. (GSE30029) [26]. *** p<0.001.

We examined Cdh1 expression levels in 29 samples of newly diagnosed AML patients. The leukemic blasts analyzed were obtained both from bone marrow (BM; 17/29) and peripheral blood (PB; 12/29) (Table 1). Except for one, primary AML cells showed a strong decrease of Cdh1 in all samples compared to normal PB CD34+ control samples (Figure 1A–1C, p<0.001). In 4 of the samples (#18, #21, #20, #15), this decrease was greater than 10-fold (Figure 1A). The decrease of Cdh1 expression was similar in blasts from BM and PB. No correlation between patient data, such as age, gender, cytogenetics, mutations, or FAB subtype and Cdh1 expression could be detected (Table 1). We also analyzed the Cdh1 expression of AML cell lines NB4 and HL-60 and found that Cdh1 in both AML cell lines was much lower expressed and about half of what we observed in PB CD34+ control samples (Figure 1D, 1E). Therefore, we confirmed that the cell lines were comparable to primary samples.

**Table 1:** Patient characteristics

Patients	Age (Years)	Sex	FAB	Source	Blasts (%)	Cytogenetics	Mutations
AML#1	76	F	M1	PB	97	normal	
AML#2	26	F	M1	PB	86	complex	
AML#3	63	F	M1	PB	85	normal	FLT-LM, NPM1
AML#4	65	M	M1	PB	92	normal	
AML#5	21	M	M1/2	PB	99	+11	
AML#6	60	F	M2	PB	67	t(8;21)	
AML#7	67	M	M2	BM	85	inv 16	
AML#8	19	M	M3	BM	80	t(15;17)	
AML#9	63	F	M3	BM	95	t(15;17)	FLT-LM
AML#10	58	F	M3	PB	88	t(15;17), +8	
AML#11	63	M	M3	BM	99	t(15;17)	
AML#12	36	M	M4eo	BM	99	normal	FLT-LM
AML#13	28	F	M4eo	BM	90	inv16	
AML#14	46	F	M4eo	BM	70	inv16, 7q-, complex	
AML#15	58	M	tAML	BM	65	-Y	
AML#16	31	M	M4/5	BM	99	normal	
AML#17	74	M	M4/5	PB	99	20q-	NPM1
AML#18	61	M	M4/5	BM	90	normal	
AML#19	70	F	M5	BM	99	9q-	FLT-TKD, NPM1
AML#20	51	F	M5	BM	55	+14, t(1;3)	FLT3-LM
AML#21	56	F	M5	BM	90	normal	NPM1
AML#22	66	F	M5	PB	80	normal	CBL, NPM1
AML#23	68	F	M5	PB	95	t(11;19)	
AML#24	82	F	AML/MDS	BM	55	normal	RUNX1
AML#25	64	M	NOS	BM	99	normal	FLT3-LM, NPM1
AML#26	66	F	NOS	BM	99	normal	FLT-LM, NPM1
AML#27	67	M	NOS	PB	80	t(6;9)	FLT-LM
AML#28	31	M	NOS	BM	99	normal	
AML#29	46	F	M4	PB	95	normal	FLT-LM, NPM1

F: female, M: male

FAB: French-American-British classification, NOS: not otherwise specified, MDS: myelodysplastic syndrome

BM: bone marrow, PB: peripheral blood

**Figure 2:**
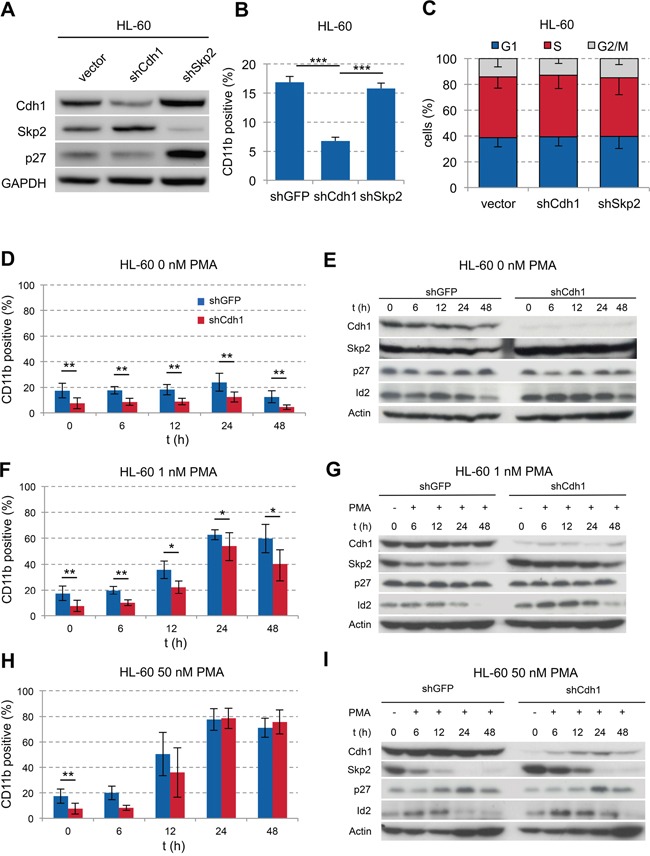
Influence of Cdh1-kd in AML cell line HL-60 on PMA-induced monocytic differentiation **A.** HL-60 cells were transduced with vectors, expressing shRNA against human Cdh1 or Skp2 mRNA and analyzed by western blot for indicated proteins. **B.** Flow cytometric detection of CD11b expression in transduced HL-60 cells presented as mean + s.d. (n=3); *** p<0.001. **C.** Cell cycle status of tranduced cells analyzed by flow cytometry following PI staining presented as mean - s.d. (n=3) **D-I.** HL-60 cells +/− Cdh1-kd were treated with increasing concentrations of PMA for up to 48 h. Expression of CD11b was analyzed by flow cytometry over time to follow differentiation and presented as mean +/− s.d. (D+F n=6, H n=3). Cells were sampled for immunoblotting to analyze Cdh1 and related target protein expression as indicated. * p <0.05, ** p<0.01.

To further investigate the low abundance of Cdh1 in primary AML cells, we reanalyzed published microarray data [26]. Results showed that Cdh1 transcription levels were not significantly different in CD34+ AML cells compared to normal CD34+ cells (Figure 1F). These findings denote that decreased Cdh1 protein expression in primary AML blasts is predominantly due to a post-transcriptional mechanism, such as gene silencing by specific microRNAs or induction of protein degradation. Indeed proteolysis of Cdh1 mediated by the ubiquitin-ligase SCF has been described in human cancer cell lines [27, 28]. Therefore, we inhibited the SCF complex by expressing a dominant-negative mutant of the core SCF subunit Cullin-1 (Cul1-N252) and found a strong increase of Cdh1 in the AML cell lines Kasumi-1 and HL-60, while there was no difference in the cell cycle profile (Figure 1G). This is consistent with SCF-dependent post-transcriptional degradation of Cdh1 as a potential underlying mechanism of low Cdh1 expression in AML. F box proteins mediate target specificity of the SCF ubiquitin ligases and it has been demonstrated that the F box protein Skp2 is frequently increased in AML [29]. Thus, elevated expression of Skp2 could be responsible for low Cdh1 expression in AML. Accordingly, we found increased Cdh1 expression upon Skp2-kd in HL-60 and NB4 myeloid leukemia cells (Figure 2A, Figure 4A). Therefore, we also analyzed the published microarray data set [26] for Skp2 expression and found a slightly lower expression of Skp2 in primary AML. Thus, the underlying mechanism of the described increase of Skp2 protein [29] may also be post-transcriptional.

### Cdh1 knockdown contributes to a differentiation block in HL-60

To evaluate the functional role of Cdh1 in AML, we performed knockdown (kd) experiments using the FAB M2 cell line HL-60 [30]. Cdh1-kd led to stabilization of its target protein Skp2 with corresponding slight decrease of p27 indicating functional knockdown (Figure 2A). In contrast, the kd of Skp2 led to stabilization of its target protein p27 and interestingly of Cdh1. There was also a significant decrease of CD11b expression in Cdh1-kd cells compared to shGFP or shSkp2 suggesting a less differentiated state or inhibition of maturation (Figure 2B).

Neither Cdh1-kd nor Skp-kd had an effect on the cell cycle profile of HL-60 cells (Figure 2C). Next, we stimulated HL-60 cells with phorbol-12-myristate-13-acetate (PMA) to induce differentiation. Without PMA stimulation the significant difference in CD11b expression remained stable over 48 hours (Figure 2D). In addition the Cdh1 target proteins Skp2 and Id2 also remained stabilized over 48 hours (Figure 2E). When using low doses of PMA at 1 nM, cells differentiated over time with and without Cdh1-kd, but the significant lower level of CD11b expression in cells depleted of Cdh1 was preserved (Figure 2F). The maximum CD11b expression was reached at 24h of 1 or 50 nM PMA stimulation. CD14 was slightly induced on HL-60 cells upon 1 nM PMA stimulation for 24h with a diminished induction due to Cdh1-kd. CD34 was only marginally expressed on HL-60 +/− Cdh1-kd with and without PMA stimulation (Supplementary Figure S2A). At the protein level the Cdh1 targets were still more stabilized upon Cdh1-kd as compared to the control, but PMA stimulation led to increased Cdh1 expression and a decline of the targets over time in line with increased differentiation (Figure 2G). When stimulated with high concentrations of 50 nM PMA, cells rapidly reached maximal CD11b- expression after 24 hours irrespective of Cdh1-kd. The initial lower CD11b level due to Cdh1-kd disappeared (Figure 2H) and Cdh1 protein expression increased with reciprocal decline of its targets (Figure 2I). Our data suggest that low Cdh1 expression contributes to the differentiation block in AML. However, stimulation with high concentrations of PMA (50 nM) led to re-expression of Cdh1, most likely due to enforced transcription of Cdh1 counterbalancing RNAi, which led to consecutive differentiation.

### Stimulation of NB4 cells with ATRA led to differentiation and cell cycle arrest

**Figure 3:**
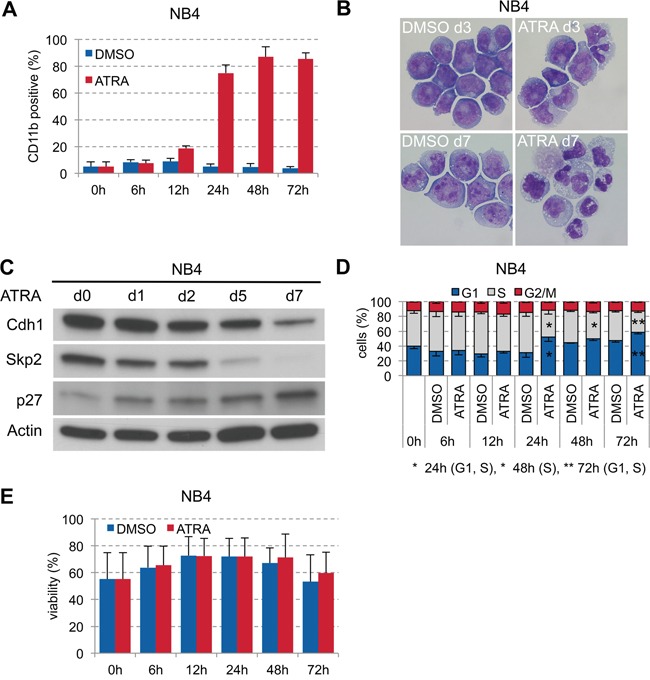
ATRA-induced granulocytic differentiation of APL cell line NB4 **A.** NB4 were plated at 0.2×10^6^ cells/ml and treated with 1 μM ATRA or DMSO for 72 h. Induction of CD11b expression to follow differentiation was analyzed by flow cytometry and presented as mean + s.d. (n=3). **B.** Differential staining of NB4 cells at 3 and 7 days of ATRA treatment compared to DMSO control to visualize differentiation to granulocytes in ATRA treated cells. **C.** NB4 cells were cultured for up to 1 week in presence of 1 μM ATRA and sampled for immunoblotting at the indicated time points. **D.** Cell cycle status of cells in (A) analyzed by flow cytometry following PI staining and presented as mean - s.d. (n=3). * p <0.05, ** p<0.01. **E.** Viability of the cells was determined by flow cytometry using dye exclusion of PI and presented as mean + s.d. (n=3).

Targeted differentiation therapy with all-trans retinoic acid (ATRA) is highly effective in acute promyelocytic leukemia (APL) with the t(15;17) translocation. Since we found low expression of Cdh1 also in APL patient samples (#13, #15, #17, #23; Table 1, Figure 1A), we investigated the role of Cdh1 in ATRA-induced differentiation in APL. Stimulation of the APL cell line NB4 with 1 μM ATRA led to strong increase of CD11b at 24 hours (Figure 3A) and granulocytic differentiation over time (Figure 3B). ATRA stimulation also caused gradual reduction of Skp2 expression until complete loss and higher p27 expression with further increase over time. Skp2 protein was assigned for degradation possibly due to ATRA-induced Cdh1 activation (Figure 3C), which only decreased at later time points when terminal granulocytic differentiation was already induced. The increase of the Cdk inhibitor p27 correlated well with cell cycle arrest in G0/G1 upon ATRA stimulation at 24 hours (Figure 3D). There was no significant difference on viability during the course of ATRA differentiation compared to the DMSO control (Figure 3E).

### ATRA could induce differentiation in APL independent of Cdh1 expression

**Figure 4:**
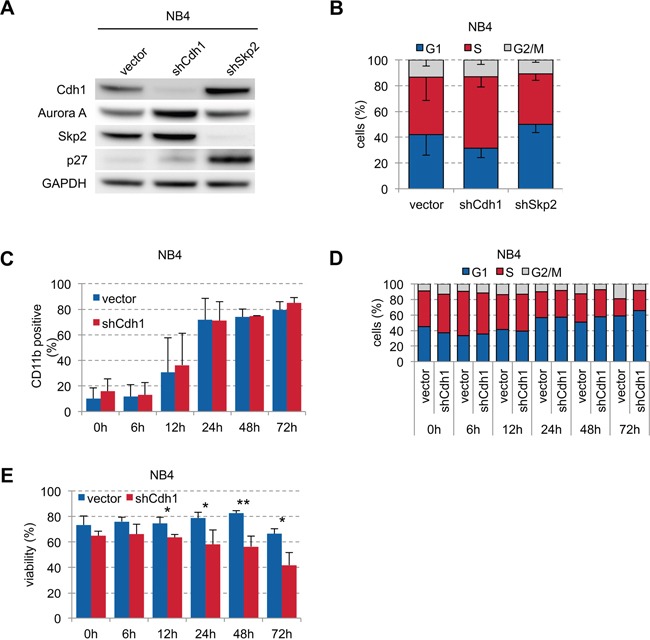
Influence of Cdh1-kd in NB4 cells on ATRA-induced granulocytic differentiation and viability **A.** NB4 cells were transduced with constructs expressing shRNA specific for human Cdh1 and Skp2 and analyzed by western blot for indicated proteins. **B.** Cell cycle status of transduced NB4 cells was analyzed by flow cytometry following PI staining and presented as mean - s.d. (n=3). **C-E.** NB4 cells were transduced with Cdh1 knockdown or empty vector and treated with 1 μM ATRA for up to 72 h. Induction of CD11b to follow differentiation (n=3), PI staining for cell cycle analysis (0-48h n=2, 72h n=1) and detection of viability (n=3) was determined by flow cytometry and presented as mean + s.d. (C, E). * p <0.05, ** p<0.01.

To further analyze the effect of Cdh1 in APL on ATRA-induced differentiation, we transduced NB4 cells with vector control and shRNAs against Cdh1 and Skp2. Cdh1-kd led to stabilization of its targets Aurora A and Skp2 without significant deregulation of p27 indicating functional kd, while Skp2-kd showed stabilization of its target p27 and also of Cdh1 (Figure 4A). Cdh1-kd led to increased proportion of S phase cells confirming previous results in other cancer cell lines [4, 31] compared to a decrease when Skp2 was depleted (Figure 4B). However, when we stimulated NB4 cells with ATRA we found that ATRA could rapidly induce differentiation (Figure 4C) and G0/G1 arrest (Figure 4D) equally well in cells with and without Cdh1-kd. This was independent of the ATRA concentration (Supplementary Figure S1) possibly due to strong transcriptional activation of inducers of differentiation which are no relevant Cdh1 targets and because APL biology depends mainly on t(15;17). However, viability of NB4 cells with Cdh1-kd was significantly reduced upon ATRA stimulation over time (Figure 4E), suggesting that low Cdh1 expression contributes to ATRA-induced cell death.

To make results between HL-60 and NB4 more comparable, we also stimulated HL-60 with 1 μM ATRA and NB4 with 1 nM PMA. ATRA had similar effects on HL-60 than PMA with significant lower expression of CD11b upon Cdh1-kd, which was preserved following stimulation with ATRA (Supplementary Figure S2B). There were also minimal, although significant differences on the very low CD14 expression following Cdh1-kd without further induction by ATRA stimulation. PMA induced rapid differentiation in NB4 cells without a significant difference in CD11b and/or CD14 expression +/− Cdh1-kd, which is comparable to the ATRA effect on NB4 (Supplementary Figure S2C). The depletion of Cdh1 did not change overall proliferation rate of HL-60 and NB4 cells (Supplementary Figure S2D).

## DISCUSSION

Cdh1 is an important regulator of proliferation and differentiation. While we found Cdh1 expression decreased in the vast majority of primary AMLs of all subtypes, Cdh1 deficiency contributes to the differentiation block in AML with maturation but not in APL. This difference is relevant, because APL is, in contrast to all other AML subtypes, highly responsive to differentiation therapy with ATRA leading in combination with other compounds, such as anthracyclines or arsenic trioxide, to high complete remission rates of the disease and long-term survival. Moreover, while low Cdh1 expression in APL did not attenuate ATRA-induced differentiation, it enhances cell death and may thereby contribute to the therapeutic effect. APL harbors a unique chromosome translocation t(15;17) resulting in the PML-RARα fusion gene which may cause the accumulation of undifferentiated promyelocytes by constitutive repression of differentiation. This is counteracted by retinoic acid, which induces myeloid differentiation [32]. Thus, in APL the transcriptional repression of differentiation genes by PML-RARα is mainly responsible for the differentiation block and modulation of APC/C^Cdh1^-dependent protein ubiquitination is ineffective. In contrast, PMA induces differentiation by transcriptional regulation and enhancement of protein ubiquitination. It has been shown that expression of Cdh1 is specifically increased during PMA-induced megakaryocyte differentiation of the bcr-abl positive myeloid leukemia cell line K562 [33], hence supporting PMA effects in non-APL AML without t(15;17), which is consistent with our data in HL-60. Cdh1-kd delays differentiation in HL-60 potentially by stabilizing Id2 (inhibitor of differentiation) without significantly altering the cell cycle profile or proliferation (Figure 2C, Supplementary Figure S2). In NB4, in contrast, Cdh1-kd resulted in increased S phase cells without affecting differentiation. ATRA may induce proliferation and differentiation of APL cells leading to the accumulation of genetically unstable differentiated cells with increased apoptosis when Cdh1 expression is low. The combination of ATRA with anthracycline-chemotherapy is standard of care in high risk APL. Our previous results demonstrated that depletion of Cdh1 prolongs S phase, promotes replication stress and genomic instability and acts synergistically on doxorubicin-induced cell cycle arrest in G2/M [25]. These observations provide an excellent explanation why the combination of ATRA und anthracyclines is highly efficient in (Cdh1 low) APL.

We found that Cdh1 is post-transcriptionally repressed in AML, which may be controlled by the SCF ubiquitin ligase. It has recently been shown that SCF^ßTrCP^ can ubiquitinate Cdh1 for degradation [28]. The overexpression of SCF subunit Skp2 has been shown to contribute to tumorigenesis in prostate cancer, breast cancer and lymphoma [34–37]. Since Skp2 protein expression is also increased in AML [29], we analyzed a potential additional role of Skp2 in Cdh1 degradation and found elevated Cdh1 levels in leukemia cells depleted of Skp2. This is an intriguing observation, since Skp2 is also a target of Cdh1. Thus, there may be an interesting feedback loop where early G1 is maintained by Cdh1-mediated degradation of Skp2 and prevention of p21/p27 degradation. In late G1, when Cdh1 is inactivated by phosphorylation and its inhibitor Emi1, Skp2 becomes stable and can degrade p21, p27 and Cdh1 leading to S phase entry and cell cycle progression. Whether Cdh1 is a direct SCF^Skp2^ target, remains to be further analyzed.

In summary, depending on differences in the microenvironment and the additional genetic background, our data are the first - to the best of our knowledge - to conclusively suggest that low Cdh1 expression may be important in AML biology by contributing to the differentiation block in non-APL AML and response to therapy in APL. It will be interesting to further analyze the function of Cdh1 in the differentiation and self-renewal of CD34+ normal hematopoietic and leukemic stem cells.

## MATERIALS AND METHODS

### Patients

We analyzed 29 samples of newly diagnosed acute myeloid leukemia (AML) patients at the Freiburg University Medical Center who gave written informed consent. Leukemic blasts were obtained from BM (17/29) and PB (12/29) specimens that contained high proportions of blast cells by Ficoll density gradient centrifugation. Detailed patient characteristics are summarized in Table 1. Normal CD34+ cells from mobilized autologous donors who gave informed consent were purified from apheresis by immunomagnetic cell separation Kit (CD34 MicroBead Kit, Miltenyi) [38].

### Microarray analysis

Microarray datasets were obtained from the open-access NIH Gene-Expression Omnibus (GEO) database. Datasets used in our analysis were GSE30029 [26].

### Western blot and antibodies

Immunoblotting has been performed as previously described [4]. Antibodies used were anti-Cdh1 (Merck, Abcam), anti-Cullin1 (Abcam), anti-Skp2 (Cell Signaling, Santa Cruz), anti-p27 (BD Bioscience, Cell Signaling), anti-Id2 (Santa Cruz), anti-Aurora A (BD Bioscience), anti-GAPDH (GeneTex) anti-Actin (Sigma) and horseradish-peroxidase-conjugated anti-mouse (Sigma, Dako) and anti-rabbit (Amersham) secondary antibodies. For densitometry of Western blots, the Gel iX imager (Intas Science Imaging Instruments, Göttingen, Germany) and LabImage 1D (Intas) were used.

### FACS analysis

Cells were labelled with RPE- or FITC-conjugated anti-CD11b antibody (Dako, AbD Serotech). As an isotype control Simultest Control γ1/γ1 (BD Pharmingen) was used. Propidium iodide staining for cell cycle analyses was performed as described previously [4]. Cells were analysed on a FACS Calibur (BD Bioscience, Franklin Lakes, USA) using the Cellquest software (BD Bioscience, Franklin Lakes, USA) or on LSR Fortessa (BD) and FACS Diva 6.2 software for acquisition. For data analysis FlowJo 7.6.5 was used.

### Plasmids

For lentiviral knockdown of Cdh1, Skp2 and GFP in HL-60 cells a previously described pLentiLox3.7 vector was used [4]. Oligonucleotides (Apara Bioscience GmbH) containing a 19 bp shRNA directed against Cdh1 (5′-GGATTAACGAGAATGAGAA-3′) and GFP (5′-GGCATCAAGGTGAACTTCA-3′), designed by our laboratory [4], and Skp2 (5′-GAGGAGCCCGACAGTGAGA-3′) [37], were annealed and ligated into HpaI/XhoI-site of pLentiLox3.7. Experiments in NB4 cells were carried out with the pLeGOhU6-G expression vector [39, 40]. The selection marker GFP was exchanged with RFP using the BamHI and EcoRI restriction sites. Oligonucleotides against Cdh1 and Skp2 were annealed and ligated into HpaI/XhoI-site of pLeGOhU6-RFP as described to obtain pLeGOhU6-RFP-Cdh1-kd. pLeGOhU6-RFP served as a control. A dominant negative Cullin1 N252 (Cul1-N252) construct that disrupts the function of the SCF complex was obtained from D. Guardavaccaro (Utrecht). The Cul1-N252 fragment was subcloned into pLeGO-iG [39] using BamHI and NotI restriction sites.

### Cell culture and lentiviral transduction

Cell lines Kasumi-1, HL-60 (gift from M. Lübbert) and NB4 (DSMZ #ACC 207) were cultured in RPMI 1640 medium (with 2 mM L-Glutamine, Gibco) supplemented with 10 % fetal bovine serum (Biochrom), 1 % penicillin/streptomycin (Life Technologies) and 1 mM sodium pyruvate (Life Technologies). Suspension cells were kept at a density between 0.1 - 0.5 × 10^6^ cells/ml. Lentiviral infections of cell lines were performed as previously described [4].

### Reagents

Phorbol-12-myristate-13-acetate (PMA, Sigma) was dissolved to 50 μM in DMSO/Ethanol (50 % each). Up to 50 nM PMA was added to the culture, while the final DMSO concentration did not exceeded 0.1 %. All-trans retinoic acid (ATRA, Sigma) was dissolved in DMSO to receive 50 mM stock solution.

### Differential staining (Pappenheim's stain)

Cells were spun onto glass slides by cytocentrifugation and stained for 5 min in May-Gruenwald's-eosin-methylene blue solution (Merck). After washing in PBS for 3 min slides were placed in diluted (1:20 in PBS) Giemsa's azur eosin methylene blue solution (Merck) for 20 min. Staining was stopped by washing with destilled water. Slides were imaged using an Axio Imager.A2 (Zeiss) with Plan Apochromat 63x/1.4 oil immersion objective.

### Statistical analysis

A two-sided Student's t-test was used for statistical analyses. Mean + standard deviation (s.d.) were ploted as indicated. P-values were defined as indicated in the figure legends * p<0.05, ** p<0.01, *** p<0.001.

## SUPPLEMENTARY FIGURES


