# Supplementary material for: Suppression of APC/CCdh1 has subtype specific biological effects in acute myeloid leukemia
Source: Oncotarget. 2016 Jun 21;7(30):48220–30. doi: 10.18632/oncotarget.10196 (PMC5217013; doi:10.18632/oncotarget.10196)
Supplement: Supplementary file 1 [file oncotarget-07-48220-s001.pdf]

Suppression of APC/C^{Cdh1} has subtype specific biological effects in acute myeloid leukemia

SUPPLEMENTARY FIGURES

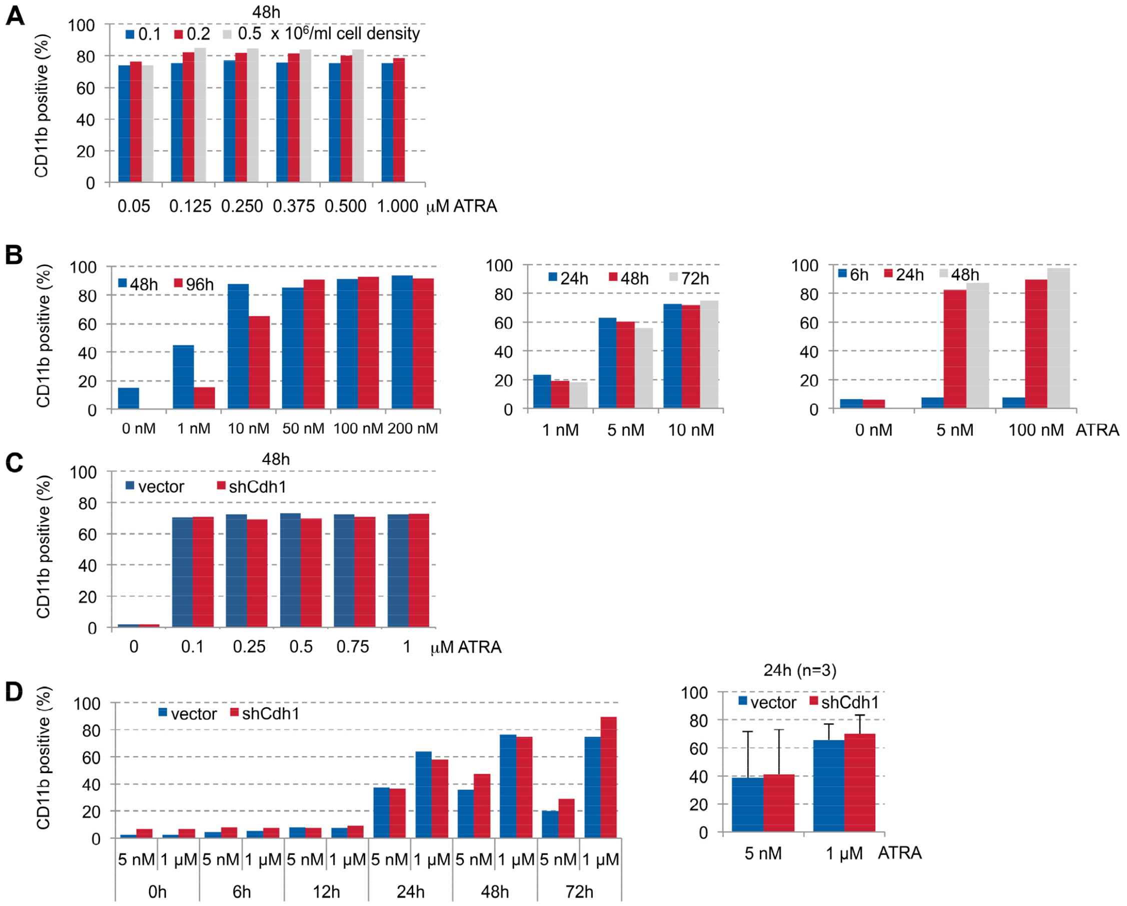

Supplementary Figure S1: Cdh1-kd has no influence on ATRA-induced differentiation independent of ATRA concentration. A. NB4 differentiation is not different at lower cell density or decreasing ATRA concentrations. **B.** The differentiation capacity is reduced at very low ATRA concentrations (1 and 5 nM). **C.** Cdh1-kd has no influence on ATRA-induced differentiation at low concentrations compared to control. **D.** The differentiation capacity is reduced at very low ATRA concentrations (5 nM) independent of Cdh1-kd.

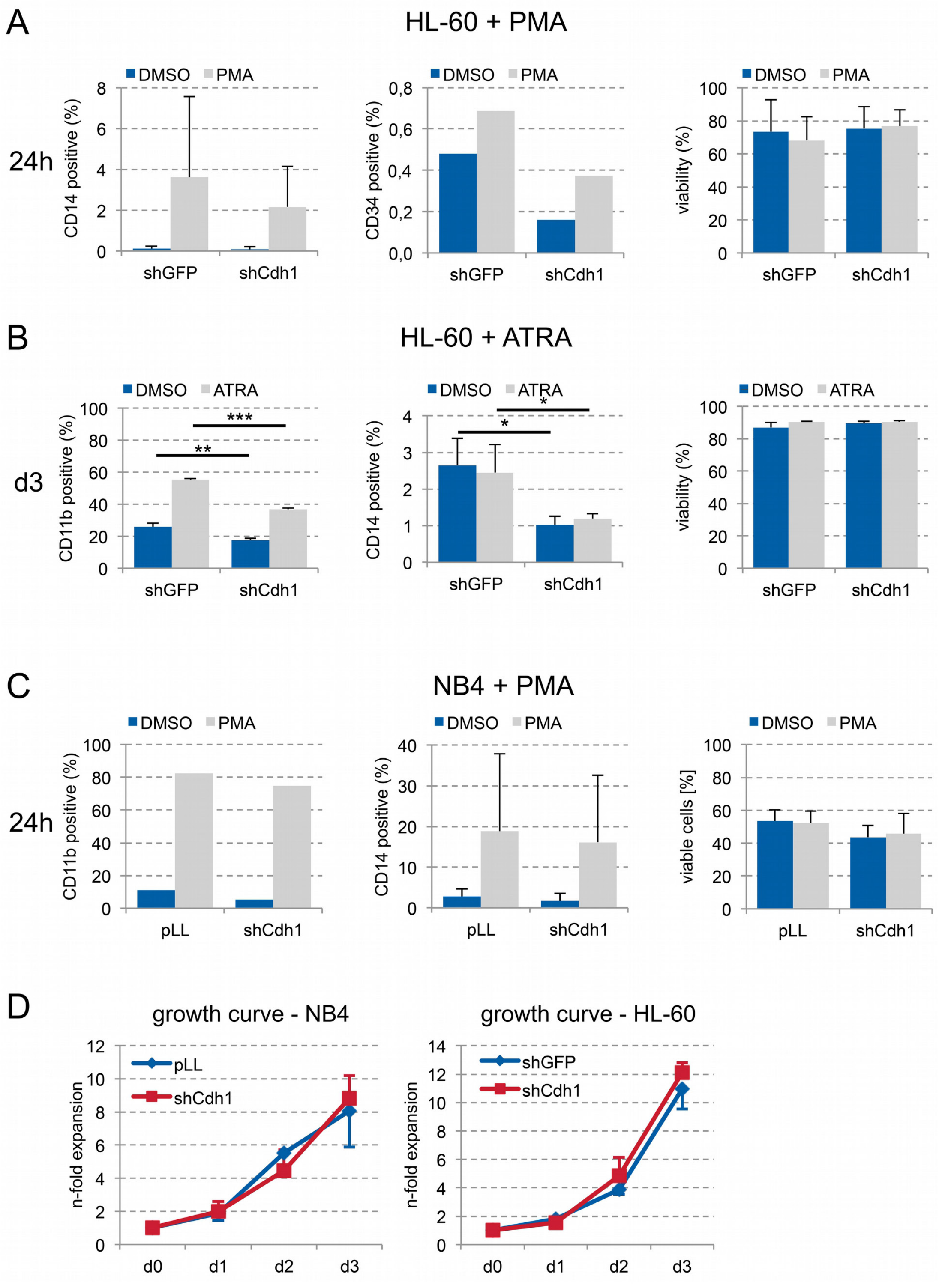

Supplementary Figure S2: Stimulation of HL-60 and NB4 with ATRA and/or PMA. A. HL-60 cells were treated for 24 h with 1 nM PMA and CD14 (n=4), CD34 (n=2) and viability using dye exclusion of PI (n=4) was determined by flow cytometry. **B.** HL-60 cells were treated for 72 h with 1 µM ATRA and CD11b, CD14 and viability was determined by flow cytometry. n=3; * $p < 0.05$, ** $p < 0.01$, *** $p < 0.001$. **C.** Differentiation of NB4 cells was induced by addition of 1 nM PMA. After 24 h CD11b (n=2), CD14 (n=3) and viability (n=3) was determined by flow cytometry. **D.** Transduced NB4 and HL-60 cells were plated at $1x10^5$ cells/ml and total cell number was determined at indicated time points (n=3).
